# Poorer survival in prenatally diagnosed trisomy 18 infants compared with postnatally diagnosed cases: a single-center study

**DOI:** 10.7717/peerj.21515

**Published:** 2026-07-08

**Authors:** Shigeki Koshida, Kentaro Takahashi

**Affiliations:** Perinatal Center, Shiga University of Medical Science, Otsu, Japan

**Keywords:** Trisomy 18, Prenatal diagnosis, Survival

## Abstract

**Background:**

Trisomy 18 is a common autosomal trisomy that is associated with high perinatal and infant mortality rates. With the widespread use of prenatal screening, many cases have been identified. In contrast, when the diagnosis occurs in the late prenatal period, that is, beyond the gestational limit for termination, or postnatally, careful planning for delivery and neonatal care is required. Emerging evidence suggests that intensive neonatal care can prolong survival and that the prenatal diagnosis may influence subsequent treatment choices. However, few studies have directly compared survival according to the timing of the diagnosis while accounting for treatment policy. Therefore, we compared survival between prenatally and postnatally diagnosed infants with trisomy 18, and examined whether diagnostic timing was associated with the selection of intensive care.

**Methods:**

A retrospective study was conducted on 35 cases of trisomy 18 identified at our center between 2005 and 2024, including 25 prenatally diagnosed cases and 10 postnatally diagnosed liveborn infants. Among the prenatally diagnosed cases, 15 resulted in live births. Survival outcomes were compared between prenatally diagnosed liveborn infants (*n* = 15) and postnatally diagnosed liveborn infants (*n* = 10), and perinatal characteristics were evaluated.

**Results:**

Among all liveborn infants, the Kaplan–Meier estimated 12-month survival rates were 8% (95% CI [0.5%–30.6%]) in the prenatal group and 40% (95% CI [12.3%–67.0%]) in the postnatal group, with poorer survival in the prenatal group (log-rank *p* = 0.02). Among liveborn infants, an intensive care policy was selected significantly less frequently in the prenatal group than in the postnatal group (33.3% [5/15] *vs.* 90.0% [9/10], *p* = 0.01). In an exploratory subgroup analysis of infants who received intensive care, the Kaplan–Meier estimated 12-month survival rates were 20% (95% CI [0.8%–58.2%]) in the prenatal group and 44% (95% CI [13.6%–71.9%]) in the postnatal group. The confidence intervals were wide, and no statistically significant difference in survival was observed between the two groups (log-rank *p* = 0.30).

**Conclusions:**

Overall survival was poorer among infants with trisomy 18 diagnosed prenatally than among those diagnosed postnatally, and this difference may be partly associated with differences in treatment policies. These findings suggest that treatment policies following prenatal diagnosis may be associated with survival duration in trisomy 18.

## Introduction

Trisomy 18 (T18) is the second most common autosomal trisomy, after Down syndrome, and is associated with an increased risk of poor perinatal and infant mortality (Cereda & Carey, 2012; Edwards et al., 1960). Advances in prenatal screening, including non-invasive prenatal testing (NIPT), have led to an increasing number of cases being diagnosed in early pregnancy. In many of these cases, pregnancy was terminated following a prenatal diagnosis of T18 before 22 weeks of gestation (Steering Committee for Prenatal Testing Approval System in Japan, 2023). In Japan, termination of pregnancy after 22 weeks of gestation is not legally permitted. Therefore, when T18 is diagnosed at or after 22 weeks of gestation, or only after birth, healthcare providers and prospective parents must carefully consider perinatal management, including delivery planning and neonatal care options. In such situations, healthcare providers are encouraged to present the latest evidence on the natural history of T18, including the potential for improved survival with intensive care, and to discuss all available care options with prospective parents. Providing accurate prognostic information is essential to support informed decisions and ensure that medical care aligns with the family’s values and preferences.

Recent reports have suggested that survival of T18 infants may be prolonged when neonatal intensive care is provided (Kosho et al., 2006; Kaneko et al., 2008; Kaneko et al., 2009; Maeda et al., 2011; Nishi et al., 2014). Although trisomy 18 is associated with multiple congenital anomalies and high mortality, current clinical practice increasingly emphasizes individualized counseling and shared decision-making rather than uniform non-intervention. Therefore, understanding outcomes according to diagnostic timing and care policy is clinically relevant for families and clinicians. Our previous study also demonstrated that intensive care could significantly improve the survival of infants with T18 (Koshida & Takahashi, 2023). Notably, we also found that infants who did not receive intensive care were more likely to have a prenatal diagnosis than those who did. As a prenatal diagnosis typically precedes decisions regarding neonatal care, it is likely that the timing of the diagnosis itself influences postnatal treatment choices and, consequently, survival outcomes. However, few studies have directly compared survival outcomes based on the timing of the diagnosis while accounting for differences in treatment policies (Burke, Field & Morrison, 2013; Cortezzo, Tolusso & Swarr, 2022; Goel et al., 2019). The effect of a prenatal diagnosis on survival outcomes in infants with T18 remains unclear.

Therefore, we compared survival outcomes between infants with T18 diagnosed prenatally and those diagnosed postnatally. We also evaluated the association between diagnostic timing and intended care policy for fetuses and neonates with T18.

## Materials & Methods

### Study design and ethical statement

This was a retrospective, single-center study conducted at Shiga University of Medical Science Hospital from 2005 to 2024. This study was approved by the Ethics Committee of Shiga University of Medical Science on December 1, 2021 (Approval No. R2021–144). Informed consent was obtained from participants using an opt-out procedure *via* the institutional website. We aimed to analyze pregnancy outcomes in fetuses prenatally diagnosed with T18 and to compare neonatal survival outcomes between liveborn infants who were prenatally diagnosed and those who were postnatally diagnosed.

### Study population

**Figure 1:**
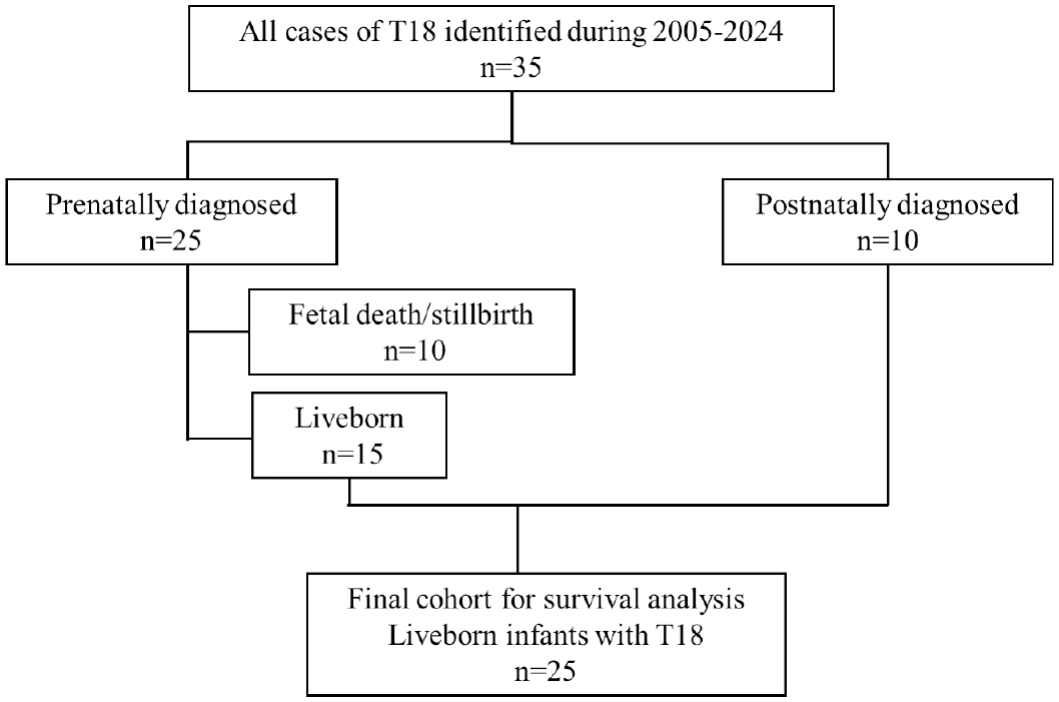
Flow diagram of the study population. Among 35 cases of trisomy 18, 25 were diagnosed prenatally, including 15 live births and 10 fetal deaths/stillbirths, and 10 were diagnosed postnatally. Survival analyses were limited to 25 liveborn infants.

A total of 35 cases of T18 were identified during the study period, including 25 prenatally diagnosed cases and 10 postnatally diagnosed liveborn infants (Fig. 1). The prenatally diagnosed group (prenatal group) consisted of fetuses diagnosed with T18 through prenatal chromosomal testing by amniocentesis. Non-liveborn cases in the prenatal group, including fetal deaths and stillbirths, were classified as prenatally diagnosed cases because trisomy 18 had already been confirmed prenatally by amniocentesis. Among the 25 prenatally diagnosed cases, 15 resulted in live births and were included in the survival analysis. The postnatally diagnosed group (postnatal group) included 10 infants diagnosed with T18 through postnatal chromosomal testing without a prior prenatal diagnosis. Therefore, the final cohort for survival analysis consisted of 25 liveborn infants. Because prenatal diagnosis may influence perinatal management decisions before birth, including fetal-oriented delivery management and neonatal resuscitation, analyses of intended care policy included all prenatally diagnosed cases regardless of live birth status.

Our medical care policy for T18 was based on a shared decision-making approach, considering the best interests of the infant while respecting parental autonomy. In this study, “intensive care” referred primarily to neonatal intensive care provided after birth; however, the intended care policy also included fetal-oriented obstetric management before birth. When intensive care was selected prenatally, fetal-oriented obstetric management was considered, including maternal hospitalization, frequent fetal monitoring, and emergency cesarean delivery for non-reassuring fetal status when appropriate, with the aim of avoiding intrauterine fetal death. In contrast, when non-intensive care was selected, expectant outpatient management was generally continued, and emergency delivery was not performed solely for fetal indications. When a fetus or infant is diagnosed with T18, we present three care policy options: palliative, restrictive, and intensive care, which guide both perinatal and neonatal management. Palliative care was defined as comfort-focused care without active life-prolonging interventions, including no delivery room resuscitation, endotracheal intubation, mechanical ventilation, surgical treatment, or cardiopulmonary resuscitation. Restrictive care was defined as selective non-intensive management that allowed limited non-invasive resuscitative support in the delivery room, including oxygen administration and bag-mask ventilation when appropriate, but excluded endotracheal intubation with mechanical ventilation, vasoactive support, and surgical procedures. Intensive care was defined as active life-prolonging management, including full delivery room resuscitation, neonatal intensive care unit (NICU) admission, endotracheal intubation with mechanical ventilation, vasoactive support, and consideration of surgical interventions, including cardiac or gastrointestinal surgery.

### Data collection

We extracted data from maternal and infant medical records and patient charts. The variables included maternal characteristics, perinatal and neonatal factors, intended care policy for the fetus/neonate, postnatal management, and outcomes. Perinatal/neonatal factors included gestational age at birth, birth weight, sex, mode of delivery, and Apgar score.

The primary outcome of this study was one-year survival among liveborn infants in the prenatal and postnatal groups. Survival status was determined based on medical records and, when necessary, follow-up with the families. An anonymized case-level dataset with variables relevant to the analyses, including diagnostic timing, live birth status, selected major anomalies, treatment category, survival time, and event status, is provided as Table S1.

### Statistical analyses

Continuous variables are presented as medians with interquartile ranges and were compared using the Mann–Whitney *U* test. Categorical variables are expressed as numbers and percentages (n [%]), and differences were assessed using Fisher’s exact test because of the small sample size and sparse cell counts. Survival outcomes were analyzed using Kaplan–Meier analysis, and the two groups were compared using the log-rank test. Statistical significance was set at *p* < 0.05. All statistical analyses were performed using SPSS software (ver. 22.0; IBM Corp., Armonk, NY, USA).

Analyses of intended care policy included all prenatally diagnosed cases, whereas survival analyses were limited to liveborn infants. For the purpose of analysis, restrictive care was categorized as non-intensive care, and infants who received either palliative or restrictive care were analyzed together as the non-intensive care group.

To assess potential temporal changes during the long study period, we descriptively summarized selected variables by era: the early era (2005–2014) and the recent era (2015–2024). Fisher’s exact test was used for exploratory categorical comparisons between eras.

Kaplan–Meier analyses were performed separately among all liveborn infants and among infants who received intensive care. Time zero for survival analysis was defined as the date of birth. Death was treated as the event. Infants who were alive at the last confirmed follow-up were censored at the date of last confirmed survival. One infant who was transferred to another hospital without subsequent follow-up information was censored at the date of transfer. Follow-up data for at least 12 months or until death were available for all remaining liveborn infants.

Because of the small sample size, results were interpreted with emphasis on effect estimates and 95% confidence intervals (CI) rather than *p*-values alone.

## Results

### Characteristics

The study cohort and patient flow are shown in Fig. 1. The final cohort for survival analysis consisted of 25 liveborn infants, including 15 prenatally diagnosed and 10 postnatally diagnosed liveborn infants.

Table 1 shows the perinatal background, care policy, and the outcomes of prenatally and postnatally diagnosed T18 cases in this study. The median gestational age at diagnosis was 28 weeks in the prenatal group, which was significantly earlier than that in the postnatal group. Prenatal testing was not routinely offered at 28 weeks in Japan; rather, the median timing reflected referral and diagnostic evaluation after fetal abnormalities were suspected during ongoing pregnancy. The gestational age at delivery was 38.4 weeks in the prenatal group and 37.5 weeks in the postnatal group, without a statistically significant difference. Birth weight *z*-scores did not differ significantly between the two groups (Table 1). Among all prenatally diagnosed cases, including fetal deaths, an intensive care policy was selected significantly less frequently than in the postnatally diagnosed liveborn group (20.0% [5/25] *vs.* 90.0% [9/10], *p* < 0.001). There were 10 fetal deaths (40%) in the prenatal group.

**Table 1:** Perinatal characteristics and intended care policy by diagnostic timing in trisomy 18 (all cases, including fetal deaths).

	**Prenatally diagnosed** (*n* = 25)	**Postnatally diagnosed** (*n* = 10)	*p*
Perinatal background			
Maternal age (years)	37.0 [34.0–39.0]	39.0 [35.3–39.0]	0.37
Parity (primiparous)	10 (40)	2 (20)	0.36
GA at diagnosis (weeks)	28.0 [26.0–32.0]	37.5 [36.8–38.8]	<0.001
GA at (still)birth (weeks)	38.4 [36.2–40.2]	37.5 [36.8–38.8]	0.76
BW at (still)birth (*z*-score)	−3.6 [−4.1 to −3.2]	−3.2 [−3.4 to −2.4]	0.06
Perinatal care policy for fetus/infant			
Intensive care	5 (20)	9 (90)	<0.001
Outcomes			
Fetal death	10 (40)	–	–

A descriptive comparison between the early era (2005–2014, *n* = 17) and the recent era (2015–2024, *n* = 18) showed no statistically significant differences in prenatal diagnosis, live birth, intensive care policy, 12-month survival, or surgical treatment (all *p* > 0.05) (Table 2).

**Table 2:** Descriptive comparison of cases by study era.

	**2005–2014** (*n* = 17)	**2015–2024** (*n* = 18)	*p*
Prenatal diagnosis	12 (71)	13 (72)	1.00
Live birth	13 (77)	12 (67)	0.71
Intensive care policy	6 (35)	8 (44)	0.73
12-month survival	3 (18)	3 (17)	1.00
Surgical treatment	5 (29)	5 (28)	1.00

### Neonatal outcomes of T18 liveborn infants

Neonatal outcomes were analyzed among liveborn infants, including 15 liveborn infants in the prenatal group and 10 infants in the postnatal group (Table 3). Among the 15 liveborn infants in the prenatal group, five received intensive neonatal care. Among liveborn infants, intensive care was selected or provided significantly less frequently in the prenatal group than in the postnatal group (33.3% [5/15] *vs.* 90.0% [9/10], *p* = 0.01). In both groups, intensive care consisted of active neonatal life-prolonging management, including delivery room resuscitation, neonatal intensive care unit (NICU) admission, respiratory support when required, and consideration of surgical treatment for major anomalies. Neonatal death (within the first 28 days of life) occurred more frequently in the prenatal group (40.0%, 6/15) than in the postnatal group (10.0%, 1/10), but the difference was not statistically significant (*p* = 0.18). Survival to hospital discharge occurred in 20.0% (3/15) of the prenatal group and 60.0% (6/10) of the postnatal group, without a statistically significant difference (*p* = 0.09). No statistically significant differences were observed between the two groups in gestational age at birth, birth weight, sex, mode of delivery, Apgar score, or surgical treatment.

**Table 3:** Liveborn infants with trisomy 18: Neonatal characteristics, care policy, and early outcomes by prenatal *vs.* postnatal diagnosis.

	**Prenatal (*n* = 15)**	**Postnatal (*n* = 10)**	***p***
GA at birth (weeks)	37.5 [36.8–40.3]	37.5 [36.8–38.8]	0.89
Birth weight (g)	1,732 [1,536–1,984]	1,893 [1,685–1,928]	0.78
Sex (male)	4 (27)	2 (20)	0.70
Mode of delivery (c/s)	5 (33)	5 (50)	0.40
Apgar score at 1 min	4 [3–4]	4 [3–5]	0.55
Apgar score at 5 min	6 [4–8]	8 [6–9]	0.06
Intensive care policy	5 (33)	9 (90)	0.01
Neonatal death	6 (40)	1 (10)	0.18
Survival at discharge	3 (20)	6 (60)	0.09
Major anomaly			
Cardiac disease	15 (100)	10 (100)	1
Esophageal atresia	4 (27)	1 (10)	0.31
Surgical treatment			
Cardiac surgery	2 (13)	4 (40)	0.13
GI surgery	2 (13)	1 (10)	0.80
Any surgery	4 (27)	6 (60)	0.10

### Survival outcomes

**Figure 2:**
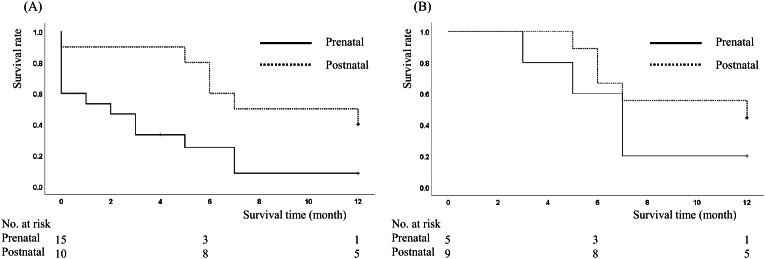
Kaplan–Meier survival curves according to diagnostic timing. (A) Survival curves among all liveborn infants with trisomy 18. The analysis included 15 prenatally diagnosed and 10 postnatally diagnosed liveborn infants. Survival differed significantly between the groups (log-rank *p* = 0.02). (B) Survival curves among infants with trisomy 18 who received intensive care. The analysis included five prenatally diagnosed and nine postnatally diagnosed infants. No statistically significant difference in survival was observed between the groups (log-rank *p* = 0.30). Numbers at risk at 0, 6, and 12 months are shown below each curve.

Kaplan–Meier survival analysis was performed among liveborn infants to compare 12-month survival between the prenatal and postnatal groups (Fig. 2). The Kaplan–Meier estimated 6- and 12-month survival rates among all liveborn infants were 25% (95% CI [6.9%–48.8%]) and 8% (95% CI [0.5%–30.6%]) in the prenatal group and 60% (95% CI [25.3%–82.7%]) and 40% (95% CI [12.3%–67.0%]) in the postnatal group, respectively. Among all liveborn infants, survival differed significantly between the prenatal and postnatal groups (log-rank *p* = 0.02) (Fig. 2A). In contrast, in the exploratory subgroup analysis restricted to infants who received intensive care, the Kaplan–Meier estimated 12-month survival rates were 20% (95% CI [0.8%–58.2%]) in the prenatal group and 44% (95% CI [13.6%–71.9%]) in the postnatal group. The confidence intervals were wide, and no statistically significant difference was observed between the groups (log-rank *p* = 0.30) (Fig. 2B).

## Discussion

Our study showed that, among liveborn infants with T18, overall survival was poorer in those diagnosed prenatally than in those diagnosed postnatally. We also found that an intensive care policy was adopted significantly less frequently in the prenatal diagnosis group than in the postnatal diagnosis group.

First, we found that, among all liveborn infants, overall survival was poorer in the prenatal diagnosis group than in the postnatal diagnosis group, with Kaplan–Meier estimated 12-month survival rates of 8% and 40%, respectively. Our study is one of the few reports to directly compare the survival outcomes based on diagnostic timing. This poorer survival in prenatally diagnosed infants is consistent with previous studies (Burke, Field & Morrison, 2013, Yamanaka et al., 2006), which included only prenatally diagnosed cases and thus did not sufficiently address the impact of diagnostic timing on survival outcomes. The interpretation of our results requires the consideration of several confounding factors. Severe fetal anomalies such as congenital heart disease may increase the likelihood of a prenatal diagnosis, whereas the level of care chosen by families and clinicians may be associated with subsequent survival. In a large population-based cohort, Cortezzo, Tolusso & Swarr (2022) showed that the factor most strongly associated with survival outcomes was the level of care selected by families and providers; however, although 18% of their cohort included postnatally diagnosed cases, outcomes were not analyzed according to diagnostic timing. Our findings suggest that prenatal diagnosis may be associated not only with postnatal care strategies but also with antepartum and intrapartum management decisions, such as fetal-oriented delivery management, which may be associated with the likelihood of live birth and subsequent survival outcomes.

Next, we found that, among liveborn infants, an intensive care policy was adopted significantly less frequently in the prenatal diagnosis group than in the postnatal diagnosis group. Our findings are consistent with those of previous studies (Acharya et al., 2021; Winn et al., 2018). One possible explanation is that fetuses with more evident anomalies or severe growth restriction tend to be diagnosed prenatally (Cereda & Carey, 2012; Cortezzo, Tolusso & Swarr, 2022; Springer et al., 2022), and clinicians and families may consequently be more likely to choose less aggressive treatment strategies. Prenatal identification may also be influenced by early ultrasound-based markers; for example, Falcon et al. (2005) reported that fetal head-to-trunk volume ratio assessed by 3D ultrasound at a median gestational age of 12 weeks characterized early growth disturbance in chromosomally abnormal fetuses. These findings support the possibility that prenatally diagnosed cases may represent a selected subgroup with more detectable fetal features, which should be considered when interpreting the external validity of our findings. However, birth weight *z*-scores did not differ significantly between the prenatal and postnatal groups in the current study. Direct comparisons of prenatal severity markers, such as fetal growth restriction or congenital anomaly severity, between the prenatal and postnatal groups were difficult because postnatally diagnosed infants, by definition, had not undergone equivalent prenatal assessment. Therefore, residual confounding by disease severity and phenotype could not be fully excluded. In contrast, infants with milder phenotypic features may escape prenatal detection and be diagnosed only after birth, potentially resulting in the more frequent adoption of intensive care policies. Goc et al. (2006) noted that T18 neonates without a prenatal diagnosis often received intensive care in NICU until cytogenetic confirmation, increasing the likelihood of aggressive interventions. Consequently, T18 infants diagnosed postnatally may have had less severe clinical presentations, which may have been associated with longer survival, independent of the chosen care policy. However, when we limited our analysis to infants who received intensive care, the Kaplan–Meier estimated 12-month survival rates were 20% in the prenatal group and 44% in the postnatal group, with no statistically significant difference between the groups (log-rank *p* = 0.30) (Fig. 2B). Because this subgroup analysis included very small numbers, the absence of statistical significance should not be interpreted as evidence of equivalent survival outcomes between the groups. Therefore, this subgroup analysis should be regarded as exploratory and hypothesis-generating, rather than as evidence that intensive care explains the survival difference between the prenatal and postnatal diagnosis groups. Taken together, the poorer survival observed in the prenatally diagnosed group may be associated with multiple factors, including differences in care policy, disease severity, phenotype, and parental decision-making processes. Therefore, treatment intent should be considered as one of several factors, rather than as the sole explanation, when interpreting survival differences between prenatally and postnatally diagnosed infants with T18.

Several limitations of the current study should be mentioned. First, the sample size was relatively small, which may limit the generalizability of the findings. Because of the small sample size, we could not perform formal multivariable adjustment for potential confounders such as anomaly severity, gestational age, birth weight, birth weight *z*-score, comorbidities, or treatment policy. Therefore, the survival comparisons were mainly based on unadjusted analyses and should be interpreted with caution. In particular, the subgroup analysis among infants who received intensive care included very small numbers and may have been underpowered to detect clinically meaningful differences in survival outcomes between the prenatal and postnatal groups. However, this study was conducted at a single institution with a consistent medical care policy for infants with T18, enabling uniform data collection and reducing the variability in treatment decisions. This setting allows for a more accurate evaluation of prognosis in relation to the timing of diagnosis and care policy.

Another limitation of this study is its retrospective design. In addition, the long study period from 2005 to 2024 may have introduced temporal changes in prenatal screening availability, parental counseling, NICU practices, surgical decision-making, and ethical approaches to trisomy 18 care. Although the exploratory era-based comparison did not show statistically significant differences in selected variables, the small sample size limited our ability to formally evaluate temporal trends. Because treatment decisions, including whether to pursue intensive care, were based on parental wishes and physician recommendations at the time of diagnosis, unmeasured confounding factors may have influenced the observed associations between the timing of diagnosis, care policy, and survival. In particular, residual confounding by disease severity and phenotype may have remained, because detailed prenatal severity markers could not be compared equivalently between the prenatal and postnatal diagnosis groups. In addition, the postnatally diagnosed group may have represented a selected subgroup of infants who survived to birth or had less apparent antenatal abnormalities, which may have affected survival comparisons. Moreover, the retrospective nature of the study limited our ability to systematically assess parental decision-making processes and clinician perspectives, both of which are crucial for understanding treatment selection and outcomes in infants with T18.

## Conclusions

In conclusion, infants with a prenatal diagnosis of T18 showed poorer survival than those with a postnatal diagnosis. These findings suggest that treatment intent may be one factor associated with prognosis in infants with T18. Clinicians should be aware that a prenatal diagnosis may influence care decisions and engage in shared decision-making to support families.

##  Supplemental Information

10.7717/peerj.21515/supp-1 Supplemental Information 1 STROBE checklist

10.7717/peerj.21515/supp-2 Supplemental Information 2 Raw data

10.7717/peerj.21515/supp-3 Supplemental Information 3 Anonymized case-level dataset of trisomy 18 cases CHD, congenital heart disease; CI, confidence interval; GA, gestational age; GI, gastrointestinal; NA, not applicable or not available. Selected major anomalies included congenital heart disease and gastrointestinal anomalies. Other anomalies were not systematically included because postnatal phenotypic evaluation was limited in infants who died shortly after birth and unavailable for non-liveborn cases. For non-liveborn cases, these variables were recorded as “Not assessed.” Restrictive care was categorized as non-intensive care for statistical analyses. Survival time is presented in months from birth. Death was treated as the event; infants alive at last confirmed follow-up or transferred without subsequent follow-up were treated as censored observations.

## Additional Information and Declarations

### Data Availability

The following information was supplied regarding data availability: The raw data is available in the Supplemental Files.
