# Supplementary material for: Poorer survival in prenatally diagnosed trisomy 18 infants compared with postnatally diagnosed cases: a single-center study
Source: PeerJ. 2026 Jul 8;14:e21515. doi: 10.7717/peerj.21515 (PMC13355608; doi:10.7717/peerj.21515)
Supplement: Supplemental Information 1 [file peerj-14-21515-s001.docx]

STROBE Statement—Checklist of items that should be included in reports of ***cohort studies***

|  | Item No | Recommendation |
| --- | --- | --- |
| **Title and abstract** | 1 | 1. Indicate the study’s design with a commonly used term in the title or the abstract   ‘Retrospectively studied’ as in the Abstract |
|  |  | 1. Provide in the abstract an informative and balanced summary of what was done and what was found   Provided in Abstract |
| Introduction | | |
| Background/rationale | 2 | Explain the scientific background and rationale for the investigation being reported  Included in the Introduction section |
| Objectives | 3 | State specific objectives, including any prespecified hypotheses  Included in the Introduction section |
| Methods | | |
| Study design | 4 | Present key elements of study design early in the paper  Included in the Study Desing part of the Methods section |
| Setting | 5 | Describe the setting, locations, and relevant dates, including periods of recruitment, exposure, follow-up, and data collection  Included in the Study Desing part of the Methods section |
| Participants | 6 | 1. Give the eligibility criteria, and the sources and methods of selection of participants. Describe methods of follow-up   Included in the Study Population part of the Methods section |
|  |  | 1. For matched studies, give matching criteria and number of exposed and unexposed   Included in the Study Population part of the Methods section |
| Variables | 7 | Clearly define all outcomes, exposures, predictors, potential confounders, and effect modifiers. Give diagnostic criteria, if applicable  Included in the Data Collection part of the Methods section |
| Data sources/ measurement | 8* | For each variable of interest, give sources of data and details of methods of assessment (measurement). Describe comparability of assessment methods if there is more than one group  Included in the Data Collection part of the Methods section |
| Bias | 9 | Describe any efforts to address potential sources of bias  Not applicable |
| Study size | 10 | Explain how the study size was arrived at  Included in the Study Population part of the Methods section |
| Quantitative variables | 11 | Explain how quantitative variables were handled in the analyses. If applicable, describe which groupings were chosen and why  Included in the Data Collection part of the Methods section |
| Statistical methods | 12 | 1. Describe all statistical methods, including those used to control for confounding   Included in the Statistical analyses part of the Methods section |
|  |  | 1. Describe any methods used to examine subgroups and interactions   Included in the Statistical analyses part of the Methods section |
|  |  | 1. Explain how missing data were addressed   Not applicable |
|  |  | 1. If applicable, explain how loss to follow-up was addressed   Not applicable |
|  |  | 1. Describe any sensitivity analyses   Not applicable |
| Results | | |
| Participants | 13* | 1. Report numbers of individuals at each stage of study—eg numbers potentially eligible, examined for eligibility, confirmed eligible, included in the study, completing follow-up, and analysed   Included in the Characteristics part of the Results section |
|  |  | 1. Give reasons for non-participation at each stage   Not applicable |
|  |  | 1. Consider use of a flow diagram   Not applicable |
| Descriptive data | 14* | 1. Give characteristics of study participants (eg demographic, clinical, social) and information on exposures and potential confounders   Included in the Characteristics part of the Results section |
|  |  | 1. Indicate number of participants with missing data for each variable of interest   Not reported |
|  |  | 1. Summarise follow-up time (eg, average and total amount)   Not applicable |
| Outcome data | 15* | Report numbers of outcome events or summary measures over time  Included in the Survival outcomes part of the Results section |
| Main results | 16 | 1. Give unadjusted estimates and, if applicable, confounder-adjusted estimates and their precision (eg, 95% confidence interval). Make clear which confounders were adjusted for and why they were included   Included in the Survival outcomes part of the Results section |
|  |  | 1. Report category boundaries when continuous variables were categorized   Included in the Survival outcomes part of the Results section |
|  |  | 1. If relevant, consider translating estimates of relative risk into absolute risk for a meaningful time period   Included in the Survival outcomes part of the Results section |
| Other analyses | 17 | Report other analyses done—eg analyses of subgroups and interactions, and sensitivity analyses  Included in the Survival outcomes part of the Results section |
| Discussion | | |
| Key results | 18 | Summarise key results with reference to study objectives  Included in the opening part of the Discussion section |
| Limitations | 19 | Discuss limitations of the study, taking into account sources of potential bias or imprecision. Discuss both direction and magnitude of any potential bias  Included in the Discussion section |
| Interpretation | 20 | Give a cautious overall interpretation of results considering objectives, limitations, multiplicity of analyses, results from similar studies, and other relevant evidence  Included in the Discussion section |
| Generalisability | 21 | Discuss the generalisability (external validity) of the study results  Included in the Discussion section |
| Other information | | |
| Funding | 22 | Give the source of funding and the role of the funders for the present study and, if applicable, for the original study on which the present article is based  Not applicable |

*Give information separately for exposed and unexposed groups.

**Note:** An Explanation and Elaboration article discusses each checklist item and gives methodological background and published examples of transparent reporting. The STROBE checklist is best used in conjunction with this article (freely available on the Web sites of PLoS Medicine at http://www.plosmedicine.org/, Annals of Internal Medicine at http://www.annals.org/, and Epidemiology at http://www.epidem.com/). Information on the STROBE Initiative is available at http://www.strobe-statement.org.
