# Supplementary material for: Poorer survival in prenatally diagnosed trisomy 18 infants compared with postnatally diagnosed cases: a single-center study
Source: PeerJ. 2026 Jul 8;14:e21515. doi: 10.7717/peerj.21515 (PMC13355608; doi:10.7717/peerj.21515)
Supplement: Supplemental Information 3 — CHD, congenital heart disease; CI, confidence interval; GA, gestational age; GI, gastrointestinal; NA, not applicable or not available. Selected major anomalies included congenital heart disease and gastrointestinal anomalies. Other anomalies were not systematically included because postnatal phenotypic evaluation was limited in infants who died shortly after birth and unavailable for non-liveborn cases. For non-liveborn cases, these variables were recorded as “Not assessed.” Restrictive care was categorized as non-intensive care for statistical analyses. Survival time is presented in months from birth. Death was treated as the event; infants alive at last confirmed follow-up or transferred without subsequent follow-up were treated as censored observations. [file peerj-14-21515-s003.docx]

**Supplementary Table S1. Anonymized case-level dataset of trisomy 18 cases included in the study**

| **Case No.** | **Year** | **Diagnostic timing** | **Live birth status** | **GA at diagnosis (weeks)** | **GA at delivery (weeks)** | **Birth weight (g)** | **Birth weight z-score** | **Sex** | **Apgar score at 1 min** | **Apgar score at 5 min** | **CHD** | **GI anomaly** | **Intended care policy** | **Surgical treatment** | **Survival time (months)** | **Event status** |
| --- | --- | --- | --- | --- | --- | --- | --- | --- | --- | --- | --- | --- | --- | --- | --- | --- |
| 1 | 2005 | Prenatal | Liveborn | 30 | 34 | 1120 | -3.68 | F | 1 | 1 | Yes | Yes | Non-intensive | No | 0 | Death |
| 2 | 2008 | Prenatal | Liveborn | 28 | 41 | 1474 | -6.28 | F | 4 | 9 | Yes | No | Non-intensive | No | 2 | Death |
| 3 | 2009 | Prenatal | Liveborn | 32 | 36 | 1822 | -2.34 | F | 5 | 9 | Yes | Yes | Intensive | Yes | 7 | Death |
| 4 | 2010 | Prenatal | Non-liveborn | 30 | 35 | 1434 | -3.19 | M | NA | NA | Not assessed | Not assessed | Non-intensive | NA | NA | NA |
| 5 | 2010 | Prenatal | Non-liveborn | 30 | 34 | 886 | -4.16 | F | NA | NA | Not assessed | Not assessed | Non-intensive | NA | NA | NA |
| 6 | 2012 | Prenatal | Liveborn | 28 | 32 | 1478 | -0.96 | F | 1 | 1 | Yes | No | Non-intensive | No | 0 | Death |
| 7 | 2012 | Prenatal | Non-liveborn | 35 | 39 | 2002 | -2.96 | F | NA | NA | Not assessed | Not assessed | Non-intensive | NA | NA | NA |
| 8 | 2012 | Prenatal | Liveborn | 27 | 37 | 1722 | -3.33 | F | 4 | 6 | Yes | No | Non-intensive | No | 0 | Death |
| 9 | 2013 | Prenatal | Liveborn | 34 | 37 | 1732 | -2.95 | F | 2 | 4 | Yes | Yes | Non-intensive | No | 0 | Death |
| 10 | 2014 | Prenatal | Non-liveborn | 26 | 27 | 734 | -1.96 | M | NA | NA | Not assessed | Not assessed | Non-intensive | NA | NA | NA |
| 11 | 2014 | Prenatal | Liveborn | 37 | 37 | 1976 | -2.55 | M | 1 | 1 | Yes | No | Non-intensive | No | 0 | Death |
| 12 | 2014 | Prenatal | Liveborn | 17 | 37 | 1490 | -3.77 | F | 3 | 6 | Yes | Yes | Intensive | Yes | 3 | Death |
| 13 | 2015 | Prenatal | Non-liveborn | 24 | 38 | 1782 | -3.30 | F | NA | NA | Not assessed | Not assessed | Non-intensive | NA | NA | NA |
| 14 | 2015 | Prenatal | Liveborn | 26 | 40 | 2265 | -3.20 | F | 3 | 3 | Yes | No | Non-intensive | No | 4 | Censored |
| 15 | 2017 | Prenatal | Liveborn | 28 | 40 | 1992 | -3.61 | M | 4 | 8 | Yes | No | Intensive | Yes | 7 | Death |
| 16 | 2017 | Prenatal | Liveborn | 33 | 41 | 1664 | -5.74 | M | 4 | 9 | Yes | No | Intensive | No | 5 | Death |
| 17 | 2019 | Prenatal | Non-liveborn | 32 | 40 | 1966 | -3.82 | M | NA | NA | Not assessed | Not assessed | Non-intensive | NA | NA | NA |
| 18 | 2020 | Prenatal | Liveborn | 27 | 39 | 2052 | -3.60 | F | 4 | 6 | Yes | No | Non-intensive | No | 0 | Death |
| 19 | 2021 | Prenatal | Non-liveborn | 33 | 39 | 1618 | -4.94 | M | NA | NA | Not assessed | Not assessed | Non-intensive | NA | NA | NA |
| 20 | 2022 | Prenatal | Non-liveborn | 24 | 39 | 1032 | -6.97 | F | NA | NA | Not assessed | Not assessed | Non-intensive | NA | NA | NA |
| 21 | 2022 | Prenatal | Liveborn | 28 | 40 | 1910 | -4.05 | F | 4 | 8 | Yes | No | Intensive | Yes | 12 | Censored |
| 22 | 2022 | Prenatal | Non-liveborn | 23 | 31 | 488 | -5.22 | M | NA | NA | Not assessed | Not assessed | Non-intensive | NA | NA | NA |
| 23 | 2023 | Prenatal | Liveborn | 27 | 37 | 1582 | -3.68 | M | 4 | 7 | Yes | No | Non-intensive | No | 3 | Death |
| 24 | 2024 | Prenatal | Liveborn | 22 | 40 | 2170 | -3.27 | F | 4 | 4 | Yes | No | Non-intensive | No | 1 | Death |
| 25 | 2024 | Prenatal | Non-liveborn | 31 | 38 | 1418 | -4.58 | F | NA | NA | Not assessed | Not assessed | Non-intensive | NA | NA | NA |
| 26 | 2007 | Postnatal | Liveborn | 41 | 41 | 2264 | -3.34 | F | 10 | 10 | Yes | No | Intensive | Yes | 12 | Censored |
| 27 | 2010 | Postnatal | Liveborn | 30 | 30 | 1050 | -2.17 | M | 3 | 6 | Yes | No | Intensive | No | 12 | Death |
| 28 | 2010 | Postnatal | Liveborn | 41 | 41 | 2240 | -3.34 | F | - | - | Yes | No | Intensive | Yes | 12 | Censored |
| 29 | 2014 | Postnatal | Liveborn | 37 | 37 | 1884 | -2.87 | F | 1 | 6 | Yes | No | Non-intensive | No | 0 | Death |
| 30 | 2015 | Postnatal | Liveborn | 37 | 37 | 1806 | -3.03 | F | 5 | 8 | Yes | No | Intensive | Yes | 12 | Censored |
| 31 | 2015 | Postnatal | Liveborn | 36 | 36 | 1917 | -1.90 | F | 4 | 6 | Yes | No | Intensive | No | 12 | Censored |
| 32 | 2019 | Postnatal | Liveborn | 37 | 37 | 1470 | -3.67 | F | 8 | 8 | Yes | Yes | Intensive | Yes | 6 | Death |
| 33 | 2019 | Postnatal | Liveborn | 37 | 37 | 1644 | -3.62 | F | 3 | 9 | Yes | No | Intensive | No | 6 | Death |
| 34 | 2012 | Postnatal | Liveborn | 36 | 36 | 1902 | -2.20 | M | 4 | 7 | Yes | No | Intensive | Yes | 7 | Death |
| 35 | 2022 | Postnatal | Liveborn | 39 | 39 | 1931 | -3.45 | F | 2 | 9 | Yes | No | Intensive | Yes | 5 | Death |

CHD, congenital heart disease; CI, confidence interval; GA, gestational age; GI, gastrointestinal; NA, not applicable or not available.

Selected major anomalies included congenital heart disease and gastrointestinal anomalies. Other anomalies were not systematically included because postnatal phenotypic evaluation was limited in infants who died shortly after birth and unavailable for non-liveborn cases. For non-liveborn cases, these variables were recorded as “Not assessed.”

Restrictive care was categorized as non-intensive care for statistical analyses.

Survival time is presented in months from birth. Death was treated as the event; infants alive at last confirmed follow-up or transferred without subsequent follow-up were treated as censored observations.
